# Histidine Promotes the Glucose Synthesis through Activation of the Gluconeogenic Pathway in Bovine Hepatocytes

**DOI:** 10.3390/ani11113295

**Published:** 2021-11-18

**Authors:** Tianyu Yang, Zhiqiang Cheng, Maocheng Jiang, Xiaoyu Ma, Osmond Datsomor, Guoqi Zhao, Kang Zhan

**Affiliations:** College of Animal Science and Technology, Yangzhou University, Yangzhou 225009, China; 18762304846@163.com (T.Y.); zqcheng1998@163.com (Z.C.); jmcheng1993@163.com (M.J.); maxiaoyu0417@163.com (X.M.); datsomorosmond@gmail.com (O.D.); gqzhao@yzu.edu.cn (G.Z.)

**Keywords:** histidine, bovine hepatocytes, gluconeogenesis

## Abstract

**Simple Summary:**

This study evaluated the effect of histidine (His) on hepatic glucose output and the expression of genes related to the gluconeogenic pathway in vitro. The results demonstrate that the supplementation of HIS can significantly improve the mRNA expression of phosphoenolpyruvate carboxykinase 1 (PCK1), phosphoenolpyruvate carboxykinase 2 (PCK2), fructose-1,6-bisphosphatase 1 (FBP1), and glucose-6-phosphatase (G6PC). Moreover, the addition of His ameliorated bovine hepatocytes glucose output. This study demonstrated that bovine hepatocytes can efficiently convert His into glucose to provide the energy required.

**Abstract:**

Histidine (His) is considered to be the first-limiting amino acid (AA) on grass silage-based diets in lactation cows, which correlate positively with lactose yield. The higher glucose requirements of lactating cows can be met through a combination of increased capacity for gluconeogenesis and increased supply of gluconeogenic precursors. However, the effect of His on the expression of gluconeogenic genes in the bovine hepatocytes is less known. Therefore, this study aimed to investigate the regulatory effect of His on the key gluconeogenic genes and glucose output in bovine hepatocytes. The addition of 0.15, 0.6, and 1.2 mM His in a medium significantly enhanced (*p* < 0.05) the viability of bovine hepatocytes. Remarkably, 1.2 mM His induced profound changes (*p* < 0.05) in the mRNA level of key genes involved in gluconeogenesis, including PCK1, PCK2, FBP1, and G6PC in vitro. Furthermore, the mRNA expression of PCK1 was significantly elevated (*p* < 0.05) by the addition of 1.2 mM His at 3, 6, 12, and 24 h of incubation. The hepatic glucose output increased (*p* < 0.05) linearly with increasing His concentration. These findings indicate that the addition of His may be efficiently converted into glucose via the upregulation of genes related to the gluconeogenic pathway.

## 1. Introduction

Histidine (His) is the first-limiting amino acids (AA) on grass silage-based diets in lactation cows, which can remarkably enhance milk yield [1]. However, low dietary His supply can decrease milk yield in lactation cows [2]. Lactose production regulates milk secretion because of high osmolarity, and it is highly correlated to milk yield [3]. Previous studies have reported that a sufficient proportion of His bypasses the rumen to increase the milk and lactose yield in lactating cows fed grass silage-based diets [4,5]. Bickerstaffe et al. (1974) demonstrated that glucose is the main synthetic precursor of lactose [6]. Therefore, the increased milk lactose yield through the addition of His suggests that supplementation with His could increase the glucose output needed to meet lactose synthesis on grass silage-based diets. Moreover, gluconeogenesis is vital to the production of milk lactose, which is a significant element of milk yield [7]. We were interested in testing whether an increase in lactose yield through His supplementation was due to gluconeogenic pathway activation, as propionate-induced gluconeogenesis has been previously explored [8]. The proportion of propionate involved in gluconeogenesis is between 50% and 60% in bovine hepatocytes [7]. However, supplementation with His increased lactose yield on grass silage-based diets in lactation cows with a low proportion of propionate in the rumen [1,4,5].

Gluconeogenesis, which occurs primarily in the liver, is essential to satisfy the metabolic glucose requirements of lactating cows and influence their milk yield [9]. The key rate-limiting enzymes involved in gluconeogenesis include the phosphoenolpyruvate carboxykinase (PCK), pyruvate carboxylase (PC), fructose-1, 6-bisphosphatase 1 (FBP1), and glucose-6-phosphatase (G6PC). In lactating cows, high rates of hepatic gluconeogenesis are associated with increased expression of phosphoenolpyruvate carboxykinase (PCK) and pyruvate carboxylase (PC) [9,10], which could promote amino acid into gluconeogenesis [11]. The conversion of fructose 1,6-diphosphate to fructose 6-phosphate is catalyzed by the enzyme FBP1. Wang et al. (2015) reported that reduced activity of the FBP1 enzyme triggers a decrease in glucose production [12]. The last step of gluconeogenesis, whereby the enzyme G6PC catalyzes glucose 6-phosphate, is necessary for the glucose release from hepatocytes [13,14]. Therefore, these enzymes are crucial in gluconeogenesis to increase glucose production. In addition, the supply of adequate glucose becomes a vital prerequisite for exploiting the genetic potential of milk synthesis [15,16]. The lactose is the primary osmoregulator of mammary water uptake to promote the milk volume [3], indicating that His may play a vital role in lactose synthesis. We hypothesized that His can enhance the expression of key genes involved in gluconeogenesis to increase glucose output in the bovine hepatocytes.

Additionally, gluconeogenesis is critical to maintaining milk lactose production [7]. Therefore, the objective of this study was to evaluate the effect of His on hepatic glucose output and the mRNA expression of phosphoenolpyruvate carboxykinase 1 (PCK1), phosphoenolpyruvate carboxykinase 2 (PCK2), PC, FBP1, and G6PC involved in gluconeogenic genes in bovine hepatocytes.

## 2. Materials and Methods

### 2.1. Isolation and Cultivation of Primary Bovine Hepatocytes

Bovine hepatocytes of three mid-lactating Holstein cows were obtained from the Institute of Animal Culture Collection and Application, Yangzhou University. The bovine hepatocytes were digested collagenase IV (Invitrogen, Shanghai, China) perfusion method, as previously defined by Liu et al. (2014) [17]. The caudate lobe of the bovine liver was acquired through surgical resection. Then, the liver was soaked three times with 20 mL solution A containing 140 mM NaCl, 10 mM N-2-hydroxyethylpiperazine-N-2-ethane sulfonic acid (HEPES), 6.7 mM KCl, 0.5 mM ethylene diamine tetraacetic acid (EDTA), and 2.5 mM glucose, with a pH 7.2 of and a temperature of 37 °C, at a rate of 120 rpm/min for 10 min and afterwards in solution B containing 140 mM NaCl, 30 mM HEPES, 6.7 mM KCl, 5 mM CaCl_2_, and 2.5 mM glucose, with a pH of 7.2 and a temperature of 37 °C, at a rate of 120 rpm/min for 10 min, finally yielding a muddy liquid. The muddy liquid containing liver was perfused with collagenase IV solution (0.1 g collagenase IV dissolved in 0.5 L of perfusion solution B, pH 7.2, at 37 °C) for the digestion of structural liver tissue. Subsequently, 50 mL fetal bovine serum (FBS; Gemini, Shanghai, China) was added to stop the digestion process. With the aid of scissors and forceps, connective tissues, fat, blood vessels and liver capsule were thoroughly eliminated. Remnant tissue fragment was cut into pieces and stained on end via a 150 μm and 75 μm mesh, sequentially. Hepatocytes were next rinsed two-fold with PBS at 4 °C, once using Red Blood Cell Lysis Buffer and suspended in a Dulbecco’s modified eagle medium/nutrient mixture f-12 (DMEM/F12) medium augmented with 10% FBS, 100 nM insulin, and 100 nM dexamethasone.

### 2.2. Cell Viability Assay

Cell Counting Kit-8 (CCK-8; Dojindo, Shanghai, China) was used according to the manufacturer’s protocol (https://dojindo.cn/#/goodsDetails?gid=582 accessed on 18 November 2021). Bovine hepatocytes were seeded into 96-well plates (5 × 10^3^ cells/well) after 12 h. Cells were then cultured in DMEM/F12 medium, free from all AA (Boster, Boster Biological Technology Co., Ltd., Pleasanton, CA, USA; control group), and in –AA medium supplemented with varying concentrations of His (at 0, 0.15, 0.6, 0.12, 0.24, 0.48, 0.96, and 1.92 mM (His-treated groups) for 24 h (*n* = 6). After incubation, cells were vigorously washed five times with 200 μL of sterile water and incubated with 200 μL DMEM/F12 supplemented with 10 μL of CCK-8 at 37 °C and 5% CO_2_ for 2 h. Auto-microplate reader was used to record each cells’ absorbance at 450 nm. Cell viability (%) = (treatment OD − blank OD)/(control OD − blank OD).

### 2.3. Quantitative RT-PCR

For mRNA expression analysis, the bovine hepatocytes were plated in 6-well plates at a density of 2 × 10^5^ cells/well with DMEM/F12 medium. Post 12 h culturing, the experiment was divided into two parts. Concentration dependence to His was investigated at 0, 0.15, 0.6, and 1.2 mM His concentration in –AA medium for 24 h. The time-dependent effect of His exposure was determined in the concentration of either 0 or 1.2 mM His for 3, 6, 12, and 24 h. A 0 or 1.2 mM His concentration in –AA mediumat 3, 6, 12, and 24 h was employed to measure time-dependent effect of His exposure. At the end of culturing, total RNA was extracted from the cells utilizing a TRIzol kit (Tiangen, Beijing, China), according to a previous study [18]. Then, the OD-1000+ Micro-Spectrophotometer was used to measure RNA purity and concentration, with RNA quality measured via electrophoresis (2% agarose gels). In our study, the optical density (OD) 260/OD280 ratio of the total RNA was determined to be 1.9, and the intensity of the 28S ribosomal RNA band was approximately twice the intensity of the 18S ribosomal RNA band in total RNA samples, indicating that total RNA was of high quality. RT Kit (Takara, Beijing, China) was used to carry out reverse transcription (RT). An amount of 1 μg total RNA and 1 × PrimeScript RT Master Mix in a final volume of 20 µL constituted RT reaction mixtures. The reaction was carried out at 37 °C for 15 min. Inactivation of reverse transcriptase was achieved by heating to 85 °C for 5 s. SYBR^®^ Premix Ex TaqTM II Kit (Takara) was employed for carrying out Quantitative Real-time PCR (qRT-PCR) assays. Constituents of qRT-PCR reaction mixture were 1 × SYBR^®^ Premix Ex TaqTM II, 0.4 μM each forward and reverse primers and 100 ng cDNA templates in a final volume of 20 µL. The reactions were carried out thus: nitial denaturation at 95 °C for 30 s, proceeded by 40 cycles at 95 °C for 5 s and 60 °C for 30 s. Prior to qRT-PCR for samples, primers were designed to span exon–exon junctions, where possible, and were evaluated for dimer formation by generating melt curves following amplification to verify the presence of a single product, according to a previous study. Table 1 shows the primers utilized. The reaction of a negative control without the cDNA sample was executed. RefFinder (http://blooge.cn/RefFinder/ accessed on 18 November 2021), including Normfinder, geNorm, and the comparative ΔCT method, were used to select the first-rank reference gene (ACTB, and GAPDH) by determining the candidate genes’ ranking. The final ranking was calculated by assigning a suitable weight value to each gene, and the geometric mean of their weight values for the overall final ranking was confirmed. A higher expression stability was indicated by a lower gene geomean of ranking value. Eventually, GAPDH was screened for subsequent study. In addition, GAPDH is already known to be suitable for bovine hepatocytes [19]. The quantitative PCR outcomes were examined by means of the 2^−ΔΔCt^ method for calculating the fold changes in the level of mRNA of targeted genes [20]. All trials were performed 3 times.

### 2.4. Quantification of Hepatic Glucose Output

For hepatic glucose output, the bovine hepatocytes were plated in a 10 cm dish at a density of 2 × 10^6^ cells/well with DMEM/F12 medium. Bovine hepatocytes supplemented with 0, 0.15, 0.6, and 1.2 mM His concentration in –AA medium for 24 h. After culture, the medium was substituted with 1 mL of glucose-free DMEM without phenol red and supplemented with 10 mM lactate and 1 mM sodium pyruvate. After culturing for an extra 2 h, the glucose level in the medium was determined by the kit (Applygen, E1011, Beijing, China). Protein concentrations were determined using a BCA kit (Beyotime, Beijing, China), according to manufacturer’s protocols. The glucose output level was normalized to that of the concentration of cells protein.

### 2.5. Statistics

Data generated were analyzed by a one-way analysis of variance (ANOVA) with least significant differences (LSD) test employed for post hoc correction for multiple comparisons of treatment means using the SPSS 16.0 software (SPSS Inc.; Chicago, IL, USA). Statistical significance was set at *p* < 0.05.

## 3. Results

### 3.1. Assessment of Cell Growth

**Figure 1:**
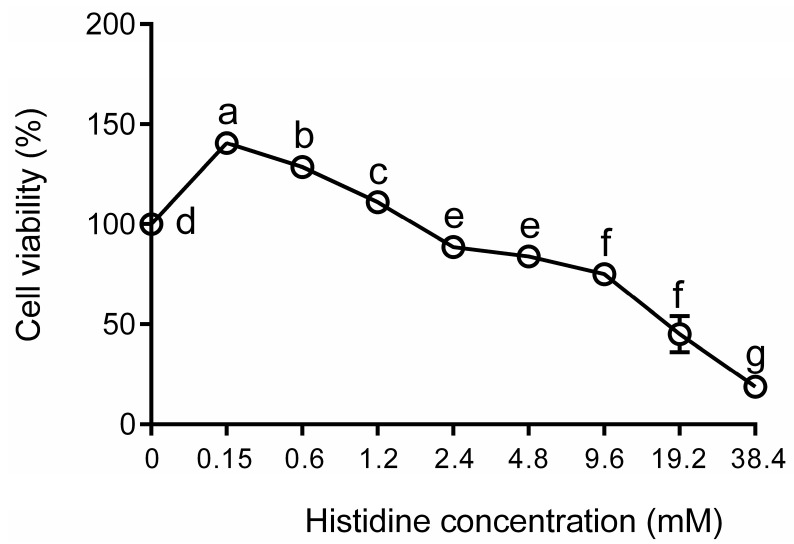
The effects of His on bovine hepatocytes. The bovine hepatocytes were incubated with 0 (control group), 0.15, 0.6, 1.2, 2.4, 4.8, 9.6, 19.2, and 38.4 mM His for 24 h. Data are presented as the means ± SEM (*n* = 6). Means at the different concentration of His with different letters (a–g) differ significantly for treatment effect.

The dose–response curves of the effect of His on bovine hepatocytes are reported in Figure 1. In particular, the number of viable cells reach (*p* < 0.05) a maximum of 1.2 mM of His. Using as a criterion proliferation ≥100% compared with the negative control, we selected three doses for His for the subsequent experiments. The current results demonstrate that the addition of 0.15, 0.6, and 1.2 mM His in vitro is able to increase the proliferation of bovine hepatocytes.

### 3.2. Effects of Increasing His Concentration and Time Point on the Expression of Key Genes Encoding Gluconeogenic Pathway

**Figure 2:**
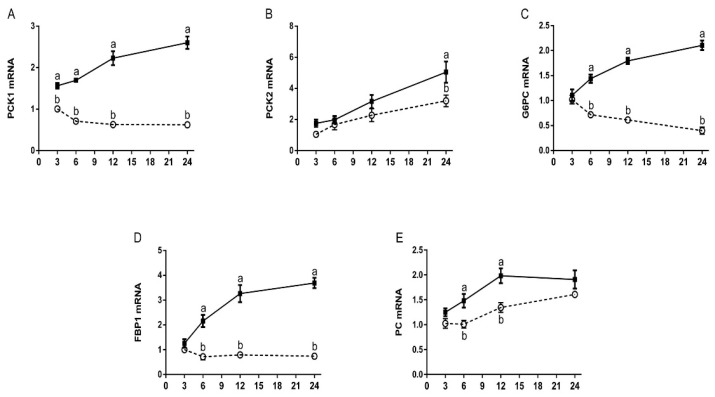
Effects of His on the mRNA levels of key genes involved in gluconeogenesis pathway. Bovine hepatocytes were exposed to either 0 (dotted line, open circle) or 1.2 mM His (solid line, solid square) for the interval indicated time and harvested for mRNA level analysis. qRT-PCR analysis of (**A**) mitochondrial phosphoenolpyruvate carboxykinase 1 (PCK1), (**B**) mitochondrial phosphoenolpyruvate carboxykinase 2 (PCK2), (**C**) glucose-6-phosphatase (G6PC), (**D**) fructose-1,6-bisphosphatase 1 (FBP1), and (**E**) pyruvate carboxylase (PC) in bovine hepatocytes. GAPDH was used as an internal reference gene. Data shown are means ± SEM of six independent experiments. Different lowercase letters indicate significant differences.

The expression of key genes involved in gluconeogenic pathway, including PCK1, PCK2, PC, G6PC, and FBP1 were investigated using qRT-PCR (Figure 2 and Table 2). The addition of 1.2 mM His markedly upregulated (*p* < 0.05) the mRNA levels of PCK1, PCK2, G6PC, and FBP1, but the expression of PC mRNA was not significantly altered (*p* < 0.05) by any of the concentrations tested, relatively to the absence of His. At 3, 6, 12, and 24 h of incubation, His markedly upregulated (*p* < 0.05) the expression of PCK1 mRNA compared with the absence of His. At 3, 6, and 12 h of incubation, His had no effect on the mRNA expression of PCK2 (*p* < 0.05). Conversely, in the treatment of 24 h incubation, the upregulation of PCK2 was observed (*p* < 0.05) with 1.2 mM His incubation. At 6 and 12 h of incubation, His significantly enhanced the expression of PC mRNA (*p* < 0.05), but the response to His dissipated by 24 h so that a lack of effect (*p* < 0.05) was observed for 24 h. At 6, 12, and 24 h of incubation, His significantly upregulated (*p* < 0.05) the expression of G6PC and FBP1 mRNA compared with the absence of His.

### 3.3. Histidine Enhances Glucose Production in Bovine Hepatocytes

**Figure 3:**
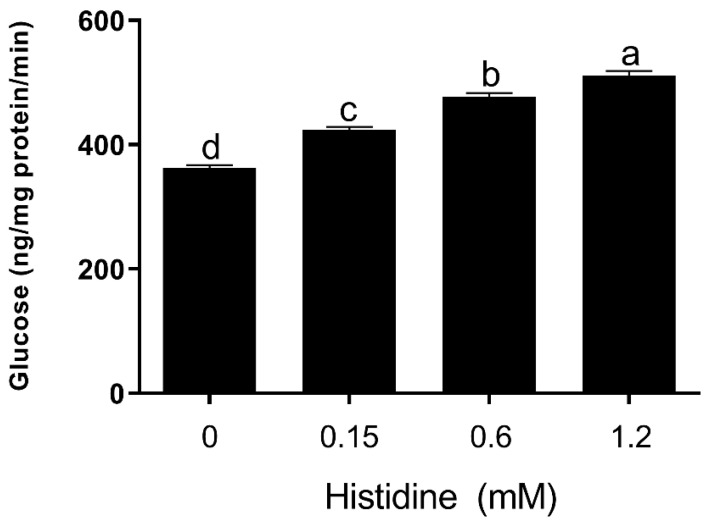
The rates of hepatic glucose output in the absence or presence of different concentrations of His. Data are presented as the means ± SEM (*n* = 6). Means at the different concentration of His with different letters (a–d) differ significantly for treatment effect.

Our discovery that His can increase the pivotal gene involved in gluconeogenesis suggests that the hepatic glucose output might be increased in bovine hepatocytes. We next investigated the direct effect of His on glucose output in bovine hepatocytes. Notably, the basal hepatic glucose output, which was determined by measurement of glucose contents released into culture media, was significantly increased (*p* < 0.05) in hepatocytes treated with His compared to the control group in Figure 3.

## 4. Discussion

Hepatic metabolism responds to increased glucose demand at the onset of lactation by activating gluconeogenesis [7]. Supplementation with 2–6.5 g/d His increased lactose yield on grass silage-based diets in lactation cows with a low proportion of propionate in the rumen [1,4,5]. Although previous studies have focused on the classical gluconeogenic pathway in bovine hepatocytes supplemented with propionate, the effect of His on the expression of gluconeogenic genes in the bovine hepatocytes is less known. The rumen fermentation pattern has been characterized by a small molar proportion of propionate in total volatile fatty acid (VFA), and enhancing the molar proportion of propionate had proved difficult in lactating cows fed silage-based diets [21]. In these diets, some of the AA are catabolized to participate in the gluconeogenesis for lactose synthesis [21]. A previous study demonstrated that when lactating cows are fed grass silage based-diets, His triggers an increase in milk and lactose yield as it becomes the first limiting AA [4]. Therefore, His may be the main gluconeogenesis precursor utilized for gluconeogenesis when lactating cows are fed grass silage-based diets.

His regulation of cell growth has been reported in bovine mammary epithelial cells [22]. Therefore, it was not surprising to observe that concentrations of 0.15, 0.6, and 1.2 mM His were able to increase the proliferation of bovine hepatocytes. Our results indicate that bovine hepatocytes proliferation was dependent on the dose of His supplementation, and cell proliferation was reduced at higher doses (2.4–38.4 mM of His), thereby in agreement with data from Gao et al. (2017) [22]. Therefore, the decrease in cell proliferation may be a consequence of cytotoxicity.

The gluconeogenic pathway includes four key rate-limiting enzymes, namely PCK, PC, FBP1, and G6PC. The enzyme encoded by PC catalyzes the formation of oxaloacetate from pyruvate in mitochondria. Oxaloacetate deficiency inhibits the tricarboxylic acid cycle and is often associated with negative energy balance and ketosis in dairy cows [23]. The increase of PC mRNA expression level correlates positively with the addition of enzyme activity in dairy cows [9]. The mRNA expression of PC is boosted to respond to more oxaloacetates from glycogenic AA and lactate as a result of increased glyconeogenesis during postpartum dairy cows [9]. Our results are in accordance with a present report that found an increase in time from 6 to 12 h significantly increased the expression of PC with 1.2 mM His, compared with the control group. The enzyme encoded by PCK1 and PCK2 catalyzes the formation of phosphoenolpyruvate from oxaloacetate in the cytosol and mitochondrion, respectively. Cytosolic PCK1 catalyzes the production of phosphoenolpyruvate from oxaloacetate, which is a crucial reaction in gluconeogenesis. In the present study, the mRNA expression of PCK1 increased through supplementation with His. Our data prove that only in the treatment of 24 h incubation, His enhanced the expression of PCK2, compared to the control group. Previous research reported that the entry of AA into gluconeogenesis is adjusted by the enzyme encoded by PC and PCK1, rather than PCK2, because the formation of phosphoenolpyruvate from AA requires the independent synthesis of NADH in the cytosol [7]. The present data seem to support our hypothesis that at lower doses, His is utilized for gluconeogenesis through the upregulation of the key gluconeogenic genes. The increasing PC and PCK1 mRNA, as a result of increasing His concentration, led to an increased intracellular phosphoenolpyruvate concentration which satisfied the next gluconeogenesis. These results indicate that His enhances the expression of PCK1 and PC, which convert pyruvate to phosphoenolpyruvate, and plays a critical role for the gluconeogenic pathway in bovine hepatocytes.

The enzyme FBP1 catalyzes the reactions in the common gluconeogenic pathway from FBP1 to fructose 6-phosphate (F6-P). Our data demonstrate that His significantly induces the expression of FBP1 in bovine hepatocytes, suggesting that His can induce more fructose 6-phosphate production for glucose synthesis in bovine hepatocytes. The enzyme encoded by the G6PC gene catalyzes the last step of gluconeogenesis to release glucose [14], at which point glucose is dephosphorylated and transported out of the hepatocyte. The expression of G6PC mRNA is transcriptionally regulated, in non-ruminants, by glucose, glucocorticoids, cyclic adenosine monophosphate (AMP), and insulin [24,25]. It is worth noting that His can significantly enhance the hepatic glucose output in hepatocytes. This is consistent with the expression of G6PC, in which the upregulation of G6PC expression promotes hepatic glucose output. Our data demonstrate that His promotes glucose concentration through the activation of the gluconeogenic pathway in bovine hepatocytes.

## 5. Conclusions

Our investigation reveals a novel role of His in inducing the expression of key gluconeogenic genes and increasing glucose output, which can efficiently convert His into glucose to meet the energy requirement in bovine hepatocytes.

## Figures and Tables

**Table 1:** Primers for real-time PCR analyses.

Gene	Primer Sequence, 5′ to 3′	Accession Number	Source
PCK1	F: 5 AGGGAAATAGCAGGCTCCAGGAAA 3 R: 5 CACACGCATGTGCACACACACATA 3	NM_174737.2	Zhang et al., 2016 [8]
PCK2	F: 5 TGACTGGGCAAGGGGAGCCG 3 R: 5 GGGGCCACCCCAAAGAAGCC 3	NM_001205594.1	Zhang et al., 2016 [8]
PC	F: 5 CCACGAGTTCTCCAACACCT 3 R: 5 TTCTCCTCCAGCTCCTCGTA 3	NM_177946.4	Zhang et al., 2016 [8]
G6PC	F: 5 TGATGGACCAAGAAAGATCCAGGC 3 R: 5 TATGGATTGACCTCACTGGCCCTCTT 3	NM_001076124.2	Zhang et al., 2016 [8]
FBP1	F: 5 ATAGAGAAGGCAGGAGGAAT 3 R: 5 CAGGAACTCAGTCACATCTT 3	NM_001034447	Zhang et al., 2016 [8]
GAPDH	F: 5 GGGTCATCATCTCTGCACCT 3 R: 5 GGTCATAAGTCCCTCCACGA 3	NM_001034034	Gong et al., 2018 [18]

F, forward; R, reverse. NM: mRNA RefSeq; PCK1: Mitochondrial phosphoenolpyruvate carboxykinase 1; PCK2: mitochondrial phosphoenolpyruvate carboxykinase 2; G6PC: glucose-6-phosphatase; FBP1: fruc-tose-1,6-bisphosphatase 1 (FBP1); PC: pyruvate carboxylase.

**Table 2:** The expression of genes involved in the gluconeogenesis pathway in bovine hepatocytes incubated with 0 (control), 0.15, 0.6, and 1.2 mM His in vitro. (*n* = 6).

	Treatment ^1^					
Symbol	Control	0.15 mM	0.6 mM	1.2 mM	SEM	*p*-Value
PCK1	1.00 ^c^	1.44 ^c^	2.38 ^b^	3.11 ^a^	0.19	<0.001
PCK2	1.01 ^d^	1.86 ^c^	4.00 ^b^	5.73 ^a^	0.40	<0.001
G6PC	1.00 ^b^	1.34 ^ab^	1.67 ^a^	1.78 ^a^	0.10	0.021
FBP1	1.00 ^b^	1.32 ^b^	3.73 ^ab^	6.47 ^a^	0.64	0.002
PC	1.00	1.27	1.24	0.91	0.51	0.34

^a,b,c,d^ Means in the same row with different superscripts differ significantly for treatment effect (*p* < 0.05). ^1^ Bovine hepatocytes were cultured in DMEM/F12 medium devoid of all AA in the absence of His (control group), in the presence of 0.15 mM His, in the presence of 0.6 mM His, or in the presence of 1.2 mM His for 24 h. PCK1: Mitochondrial phosphoenolpyruvate carboxykinase 1; PCK2: mitochondrial phosphoenolpyruvate carboxykinase 2; G6PC: glucose-6-phosphatase; FBP1: fruc-tose-1,6-bisphosphatase 1 (FBP1); PC: pyruvate carboxylase.

## Data Availability

Not applicable.
